# Burden of thyroid cancer in the older adult aged 60 and over from 1990 to 2021 and forecast of the situation in 2030

**DOI:** 10.3389/fonc.2025.1626901

**Published:** 2025-09-29

**Authors:** Hanxu Fang, Yongshuai Bian, Haoran Yang, Xi Shen, Yaqi Li, Shuling Yue, Shuyu Xiao, Yuzhen Lu, Qingzeng Qian, Fuhai Shen

**Affiliations:** ^1^ Hebei Province Key Laboratory of Occupational Health and Safety for Coal Industry, School of Public Health, North China University of Science and Technology, Tangshan, Hebei, China; ^2^ Affiliated Hospital, North China University of Science and Technology, Tangshan, Hebei, China; ^3^ Tangshan Center for Disease Control and Prevention Food Hygiene Institute, Tangshan, Hebei, China

**Keywords:** thyroid cancer, GBD database, age-related differences, sex-related, differences SDI regional disparities, time-series forecasting

## Abstract

**Background:**

This article aims to analyze the trends in global thyroid cancer mortality rate (ASMR) and disability adjusted life years (DALYs) from 1990 to 2021, and predict changes in disease burden by 2030. Through global data comparison and analysis, this study explores the impact of gender, age, and socioeconomic development level (SDI) on thyroid cancer mortality and disease burden.

**Method:**

This study is based on data from the Global Health Data Exchange (GHDx) and analyzes the trends of thyroid cancer deaths, age-standardized mortality rate (ASMR), and DALYs over the period from 1990 to 2021. The annual average rate of change (EAPC) of the data was analyzed using a joinpoint regression model. Additionally, stratified analyses were conducted by gender, age group, and SDI, and the burden of thyroid cancer for the year 2030 was projected.

**Result:**

Global thyroid cancer deaths increased from 2,198 in 1990 to 5,255 in 2021. Despite the rise in total deaths, ASMR remained stable at 0.07/100,000 for females and 0.05/100,000 for males. DALYs increased from 61,815 to 144,955, with an average annual growth rate of 0.38%. The burden on men rose significantly, while the increase was slower for women. Regions with higher SDI showed a slight decrease in burden, whereas low-SDI regions saw an increase, with an annual growth rate of 0.76%. Fiji had the highest burden, with an ASMR of 0.25 and AS-DALYs of 6.07. Female mortality and disease burden were higher than in males, particularly among those aged 85 and above.

**Conclusion:**

The global disease burden of thyroid cancer has been increasing, particularly in the older adult. While high-SDI regions experienced a decrease in burden, low-SDI regions saw an increase. Predictions suggest that by 2030, the mortality rate and disease burden may remain stable with slow annual changes. However, attention should be given to the growing burden in low-income areas and the impact of socio-economic factors.

## Introduction

Thyroid cancer is a malignant tumor originating in the thyroid gland. As the global population continues to age, the proportion of older adult individuals steadily rises, leading to an increasing burden of thyroid cancer among this demographic (1, 2). In recent years, especially among those aged 60 and above, the incidence of thyroid cancer has significantly surged. This disease not only imposes immense physical and psychological burdens on patients but also presents a grave challenge to public health.

The incidence and mortality rates of thyroid cancer among the older adult reveal pronounced gender disparities and age-related trends, with marked variations observed across different regions (3–5). The incidence and mortality rates of thyroid cancer among the older adult exhibit significant gender disparities and age-related patterns, with notable regional variations (6–8). The prevalence and mortality rates for women are markedly higher than those for men, primarily due to the higher incidence of hypothyroidism in women, which may lead to misdiagnoses of thyroid cancer, thereby distorting the statistical distribution within the overall population. Furthermore, the aging global population, accompanied by a rising proportion of older adult individuals, further exacerbates the burden of thyroid cancer among the older demographic, particularly those aged 60 and above, where both incidence and mortality rates are experiencing an annual increase. Although high-income countries, such as those in Northern Europe and North America, report relatively low rates of incidence and mortality, this trend is gradually waning as advancements in medical technology and heightened public health awareness unfold.

However, as medical technology and public health awareness continue to advance, this trend is slowly diminishing. In contrast, in middle- and low-income countries, where there is an unequal distribution of medical resources and lower healthcare standards, the burden of thyroid cancer remains more pronounced. Therefore, it is crucial to enhance early screening and diagnosis, optimize the allocation of medical resources, and elevate public health literacy as key strategies to alleviate the burden of thyroid cancer among the older adult population.

Despite the challenges associated with early screening and diagnosis, global screening activities for thyroid cancer (such as thyroid function tests and cancer screening programs) saw a significant increase between 1990 and 2021, particularly in high-income countries and certain middle-income nations (9–11). But, early diagnosis of thyroid cancer remains challenging due to its diverse clinical manifestations, which can easily be confused with other conditions such as thyroiditis and hyperthyroidism (12–14).

We conducted an investigation into the prevalence, mortality, and disability-adjusted life years (DALY) associated with thyroid cancer among adults aged 60 and above from 1990 to 2021, examining global, regional, and national data, while considering social development levels as well as the age and gender distribution of the older adult population. Additionally, we also made projections regarding the status of thyroid cancer in 2030.

## Methods

In our analysis of the 2021 Global Burden of Disease Study, we accessed burden data from 22 regions and 204 countries and territories through the Global Health Data Exchange platform. We extracted statistics concerning thyroid cancer for adults aged 60 and above, including age-standardized incidence and mortality rates, segmented by gender, region, and different SDI categories (15–17).

These countries and regions, geographically proximate and sharing similar epidemiological characteristics, were further analyzed using the SDI, a composite indicator reflecting the socioeconomic conditions that influence health outcomes in each location. The SDI ranges from 0.005 to 1, where 1 represents the highest levels of education, per capita income, and the lowest fertility rates. The SDI is categorized into five tiers: low, low-middle, middle, high-middle, and high.

We employed the JoinPoint regression program (version 5.0.2) alongside R software (version 4.2.3) to conduct statistical analyses. Descriptive statistics were utilized to characterize the burden of thyroid cancer among adults aged 60 and above on a global scale. We compared age-standardized incidence and mortality rates for specified variables across different genders, regions, and SDI categories. The EAPC was applied to reflect the average rate or magnitude of change over the designated period. Additionally, we utilized the ARIMA time series model to forecast the projected burden of thyroid cancer in 2030.

### Statistical analysis

To illustrate the global burden of thyroid cancer among adults aged 60 and above, and to examine its regional disparities by gender as well as the variations in age-standardized incidence and mortality rates across different SDI regions—as defined by the World Health Organization’s Health Support Program—we employed data from the Global Burden of Disease Study (GBD) spanning 1990 to 2021, covering 22 regions and 204 countries and territories.

### Descriptive analysis

Initially, we conducted a descriptive analysis to delineate the distribution characteristics of thyroid cancer among adults aged 60 and above on a global scale. Through comparative groupings based on gender, region, and SDI categories, we calculated the incidence, prevalence, and mortality rates of thyroid cancer, subsequently adjusting the results for age standardization.

### EAPC analysis

We utilized the EAPC to quantify the trends in the specified variables from 1990 to 2021. The EAPC value represents the percentage change per year (whether an increase, decrease, or no change). If both the annual change estimate and its 95% confidence interval (CI) were greater than 0 (or both less than 0), we interpreted the corresponding rate as exhibiting an upward (or downward) trend.

### Time series prediction

Based on the Global Burden of Disease Study data from 1990 to 2021, we developed an ARIMA time series model to forecast the age-adjusted incidence, prevalence, mortality rates, and DALY for thyroid cancer among adults aged 60 and above in 2030.

The ARIMA model, which assumes stationarity, linearity, and white noise residuals, is chosen for its simplicity, wide applicability, and solid theoretical foundation. However, it has limitations such as its inability to capture non-linear relationships, the requirement for data stationarity, low reliability in long-term predictions, inability to handle exogenous variables directly, and potential issues with seasonality.

## Result

### Global trend of mortality

From 1990 to 2021, the number of thyroid cancer deaths worldwide increased from 2,198 to 5,255, a significant increase. Although the death toll increased, the ASMR remained unchanged, indicating that although the death toll increased, the age-standardized mortality rate did not change. The number of female deaths increased from 1,494 to 3,225, showing a significant upward trend, but the ASMR remained unchanged (0.07 per 100,000 people in 1990 and 2021), and the average annual change rate EAPC was -0.12%, indicating that the ASMR of women decreased year by year. This may be related to the change of the average age structure of women, which leads to the decline of the average mortality rate. The number of male thyroid cancer deaths increased from 704 to 2,029, which was significantly higher. The ASMR increased from 0.04 to 0.05 per 100,000, with an average annual change rate of 0.89%, indicating that the mortality rate of male population increased year by year, which may be related to the increase in the incidence of male thyroid cancer. The number of deaths in high SDI areas increased from 811 to 1,368, and the ASMR decreased from 0.07 to 0.06 per 100,000 people, with an average annual change rate of -0.47%, indicating that the mortality rate in high SDI areas is decreasing year by year. The number of deaths in high-middle SDI increased from 666 to 1,194, and the ASMR decreased from 0.07 to 0.06 per 100,000 people, with an average annual change rate of -0.48%, and the mortality rate decreased. The number of deaths in the middle SDI area increased from 434 to 1,649, and the ASMR increased from 0.04 to 0.06 per 100,000 people, with an average annual change rate of 1.18%, and the mortality rate increased. The number of deaths in low-middle SDI area increased from 197 to 782, and ASMR increased from 0.03 to 0.05 per 100,000 people, with an average annual change rate of 1.8%, and the mortality rate increased. The number of deaths in low SDI areas increased from 86 to 256, and the ASMR increased from 0.04 to 0.05 per 100,000 people, with an average annual change rate of 0.92%, and the mortality rate increased slightly. (See **Table 1**).

**Table 1:** Thyroid cancer mortality trends and ASMR analysis (1990-2021).

Category	1990		2021		1990-2021
	Death cases no. *10^2^ (95% UI)	ASMR per 100,000 no. (95% UI)	Death cases no. *10^2^ (95% UI)	ASMR per 100,000 no. (95% UI)	EAPC of ASMR no. (95% CI)
Overall	21.98 [16.42-28.18]	0.06 [0.04-0.07]	52.55 [39.14-66.53]	0.06 [0.05-0.08]	0.2 [0.16 to 0.24]
Sex					
Female	14.94 [11.17-19.23]	0.07 [0.05-0.09]	32.25 [23.29-41.64]	0.07 [0.05-0.09]	-0.12 [-0.16 to -0.08]
Male	7.04 [5.15-9.01]	0.04 [0.03-0.05]	20.29 [15.1-25.96]	0.05 [0.04-0.07]	0.89 [0.83 to 0.95]
Socio-demographic index					
High SDI	8.11 [6.12-10.24]	0.07 [0.06-0.09]	13.68 [9.77-17.34]	0.06 [0.05-0.08]	-0.47 [-0.52 to -0.42]
High-middle SDI	6.66 [4.96-8.56]	0.07 [0.05-0.09]	11.94 [8.82-15.09]	0.06 [0.04-0.08]	-0.48 [-0.55 to -0.41]
Middle SDI	4.34 [3.21-5.67]	0.04 [0.03-0.06]	16.49 [12.01-21.16]	0.06 [0.05-0.08]	1.18 [1.12 to 1.24]
Low-middle SDI	1.97 [1.42-2.64]	0.03 [0.02-0.04]	7.82 [5.84-10.13]	0.05 [0.04-0.07]	1.8 [1.76 to 1.83]
Low SDI	0.86 [0.6-1.2]	0.04 [0.02-0.05]	2.56 [1.77-3.55]	0.05 [0.03-0.07]	0.92 [0.82 to 1.02]

### Disability-adjusted life year global trend

From 1990 to 2021, the number of DALY in the world increased from 61815 to 144955, a significant increase, reflecting the aggravation of the burden of illness. The AS-DALYs increased from 1.49 per 100,000 people (95% confidence interval 1.12 to 1.9) to 16.8 per 100,000 people (95% confidence interval 1.26 to 2.14), indicating that although the absolute burden of illness increased, the burden of illness standardized by age structure increased, and the health status of the whole population deteriorated. The average annual change rate (EAPC) is 0.38% (95% confidence interval is 0.34 to 0.41), which shows that DALY rate has increased year by year in the past 30 years. Grouped by gender, the pain burden of male group increased significantly, while the pain burden of female group increased, but the change was slow. According to the social and economic development level (SDI), the burden of illness in high SDI areas decreased slightly, while the burden of illness in medium SDI and low SDI areas increased significantly, especially in low SDI areas, with an average annual change rate of 0.76%, indicating that the burden of illness was on the rise but the increase was slow (See **Table 2**).

**Table 2:** Thyroid cancer DALY trends and age-standardized DALY rates (1990-2021).

Category	1990		2021		1990-2021
	DALY cases no. *10^2^ (95% UI)	Age-standardized DALY rate per 100,000 no. (95% UI)	DALY cases no. *10^2^ (95% UI)	Age-standardized DALY rate per 100,000 no. (95% UI)	EAPC of Age-standardized DALY rate no. (95% CI)
Overall	618.15 [465.71-791.16]	1.49 [1.12-1.9]	1449.55 [1092.3-1847.47]	1.68 [1.26-2.14]	0.38 [0.34 to 0.41]
Sex					
Female	409.36 [310.59-529.07]	1.87 [1.42-2.41]	881.2 [649.92-1144.69]	1.96 [1.44-2.55]	0.1 [0.06 to 0.13]
Male	208.79 [156.82-266.81]	1.06 [0.79-1.36]	568.35 [426.8-734.4]	1.38 [1.04-1.79]	0.93 [0.87 to 0.99]
Socio-demographic index					
High SDI	205.43 [156.69-259.39]	1.91 [1.46-2.41]	325.62 [248.16-410.98]	1.75 [1.32-2.2]	-0.2 [-0.28 to -0.12]
High-middle SDI	184.67 [138.94-236.42]	1.81 [1.36-2.32]	312.64 [232.43-402.72]	1.63 [1.21-2.1]	-0.4 [-0.48 to -0.33]
Middle SDI	133.04 [98.87-172.16]	1.15 [0.86-1.5]	474.48 [351.93-608.78]	1.71 [1.27-2.2]	1.28 [1.22 to 1.35]
Low-middle SDI	64.1 [46.55-86.32]	0.9 [0.65-1.21]	246.18 [186.01-322.74]	1.54 [1.16-2.02]	1.84 [1.81 to 1.88]
Low SDI	29.75 [20.67-41.83]	1.06 [0.74-1.48]	89.11 [61.6-125.71]	1.38 [0.96-1.91]	0.76 [0.67 to 0.86]

### The country with the worst burden

**Figure 1:**
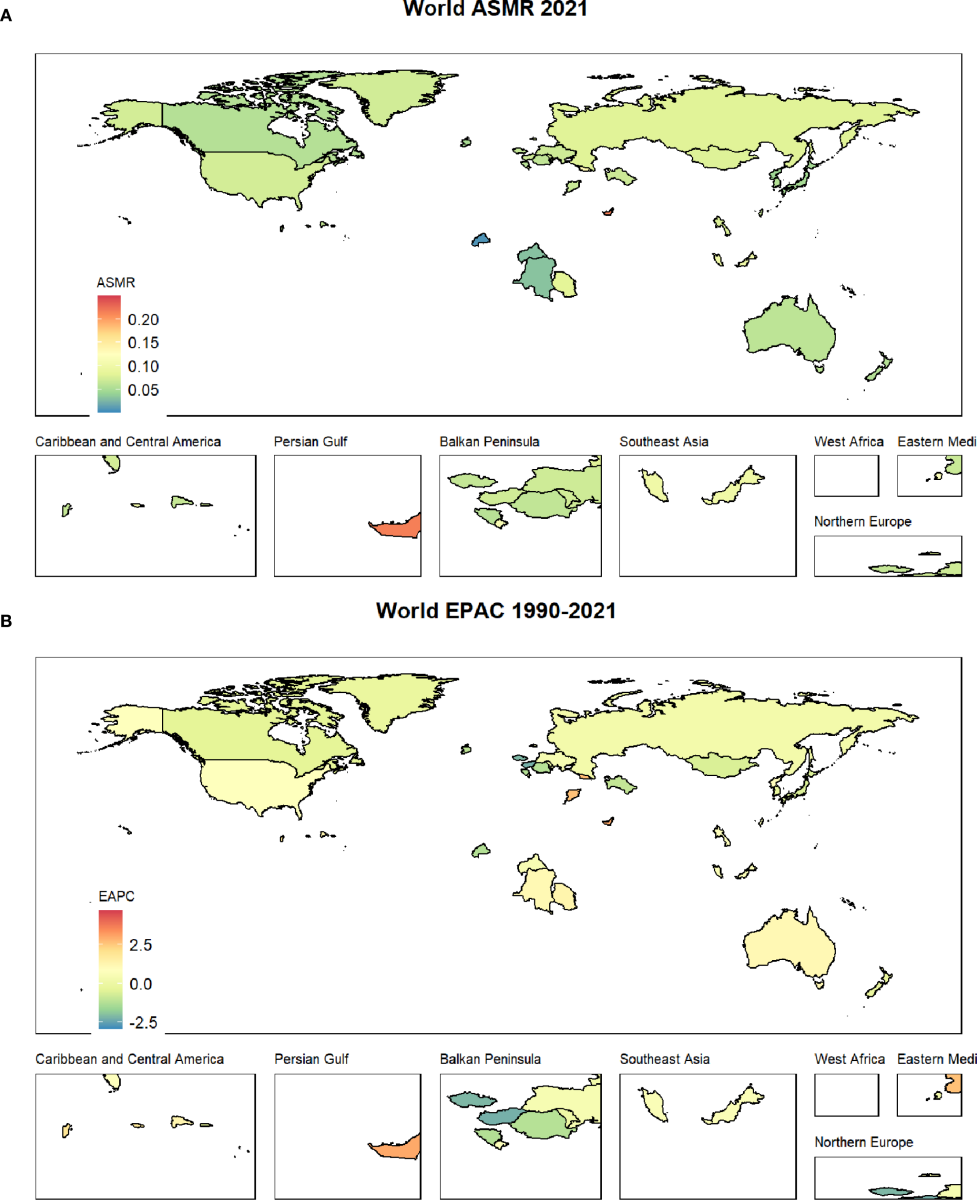
**(A)** shows the ASMR status of thyroid cancer worldwide; **(B)** displays the EAPC status of thyroid cancer from 1990 to 2021.

**Figure 2:**
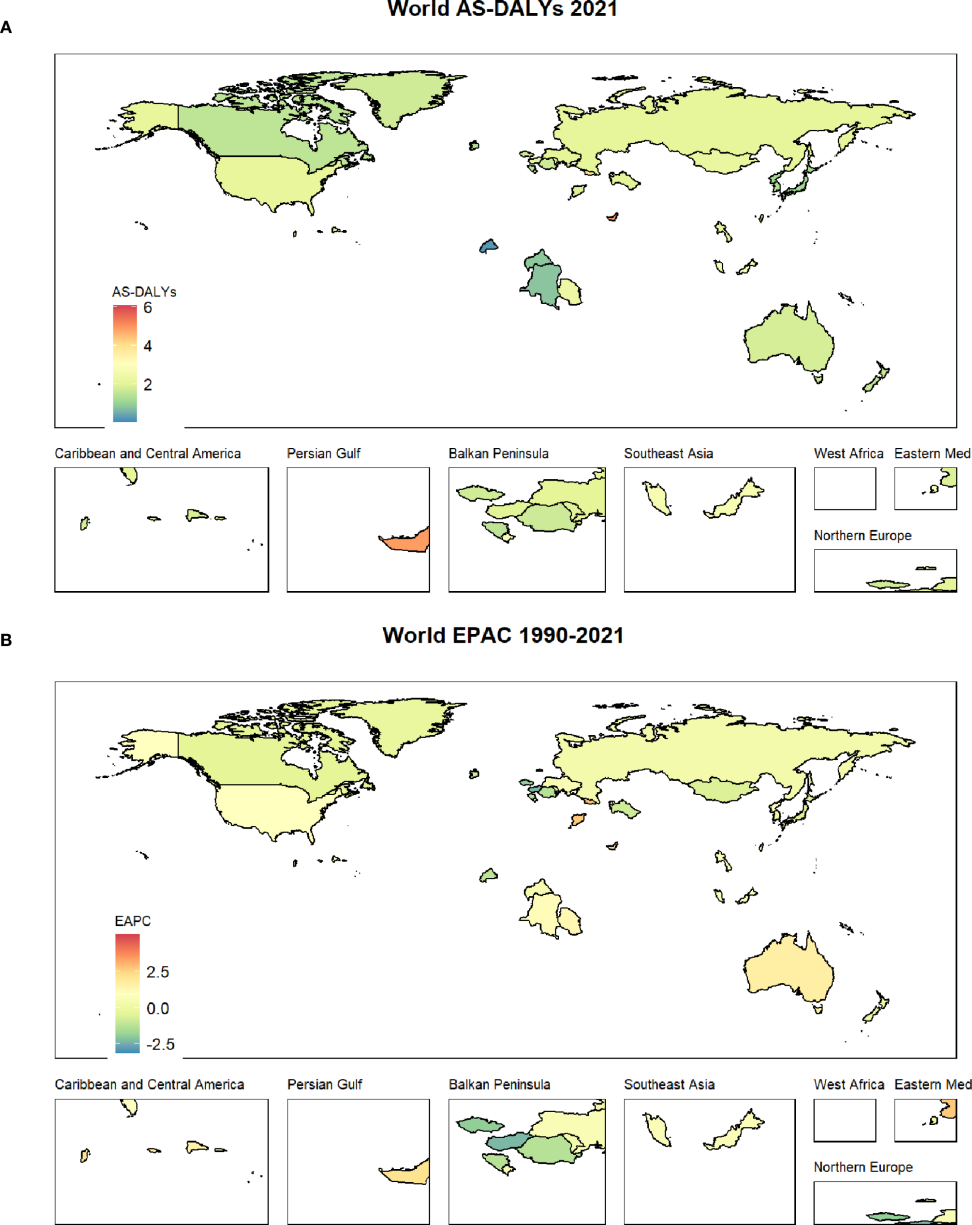
**(A)** shows the AS-DALYs status of thyroid cancer worldwide; **(B)** displays the EAPC status of thyroid cancer from 1990 to 2021.

Among the 204 countries in the world, The Republic of Fiji has the most serious burden of thyroid cancer, with the highest ASMR of 0.25(0.15-0.35) and the highest AS-DALYS of 6.07(3.76-8.98) (**Figures 1**, **2**).

### Jointpoint regression result

**Figure 3:**
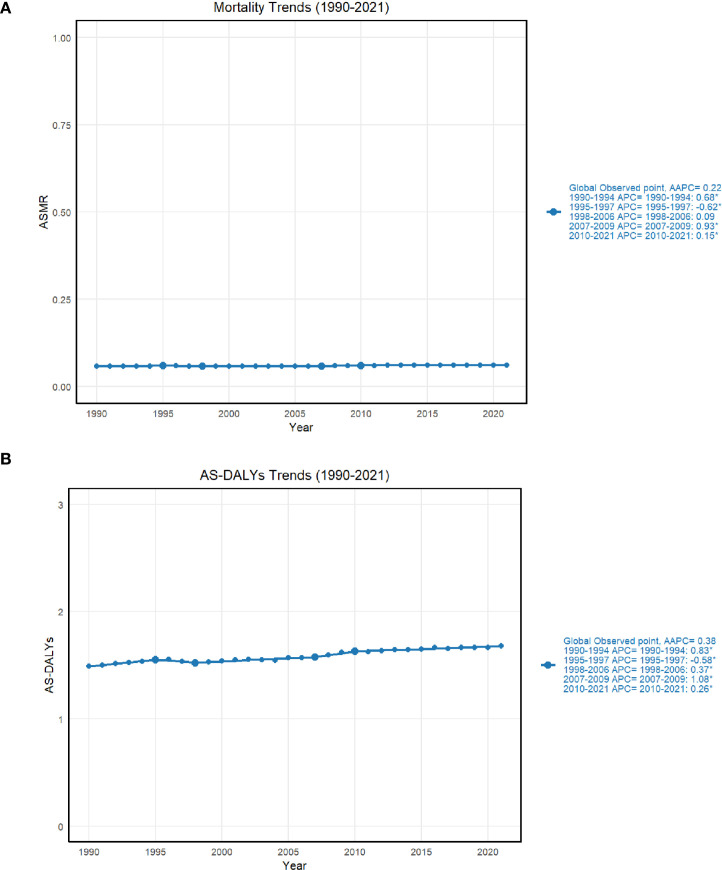
**(A)** shows the joint point regression results of ASMR in thyroid cancer; **(B)** shows the joint point regression results of AS-DALYs in thyroid cancer.

The graph analysis reveals a generally stable trend in both ASMR and AS-DALYs over time, with slight fluctuations. For ASMR, the rate remained low and relatively stable, with an overall average annual percentage change (AAPC) of 0.22%, indicating a slight upward trend in global mortality rates. Specific periods showed minor increases, such as 1990-1994 (0.68%) and 2007-2009 (0.93%), while other years like 1995–1997 saw a decline (-0.62%). The period from 2010 to 2021 showed minimal change (0.15%), pointing to overall stability with small fluctuations. Similarly, AS-DALYs exhibited a gradual increase, with an AAPC of 0.38%. Notable fluctuations occurred during 1990-1994 (0.83%) and 2007-2009 (1.08%), reflecting moderate increases, while 1995–1997 saw a slight decrease (-0.58%). The overall trend shows a modest rise in AS-DALYs, with the pace slowing in recent years (**Figure 3**).

### Trends in age and gender

**Figure 4:**
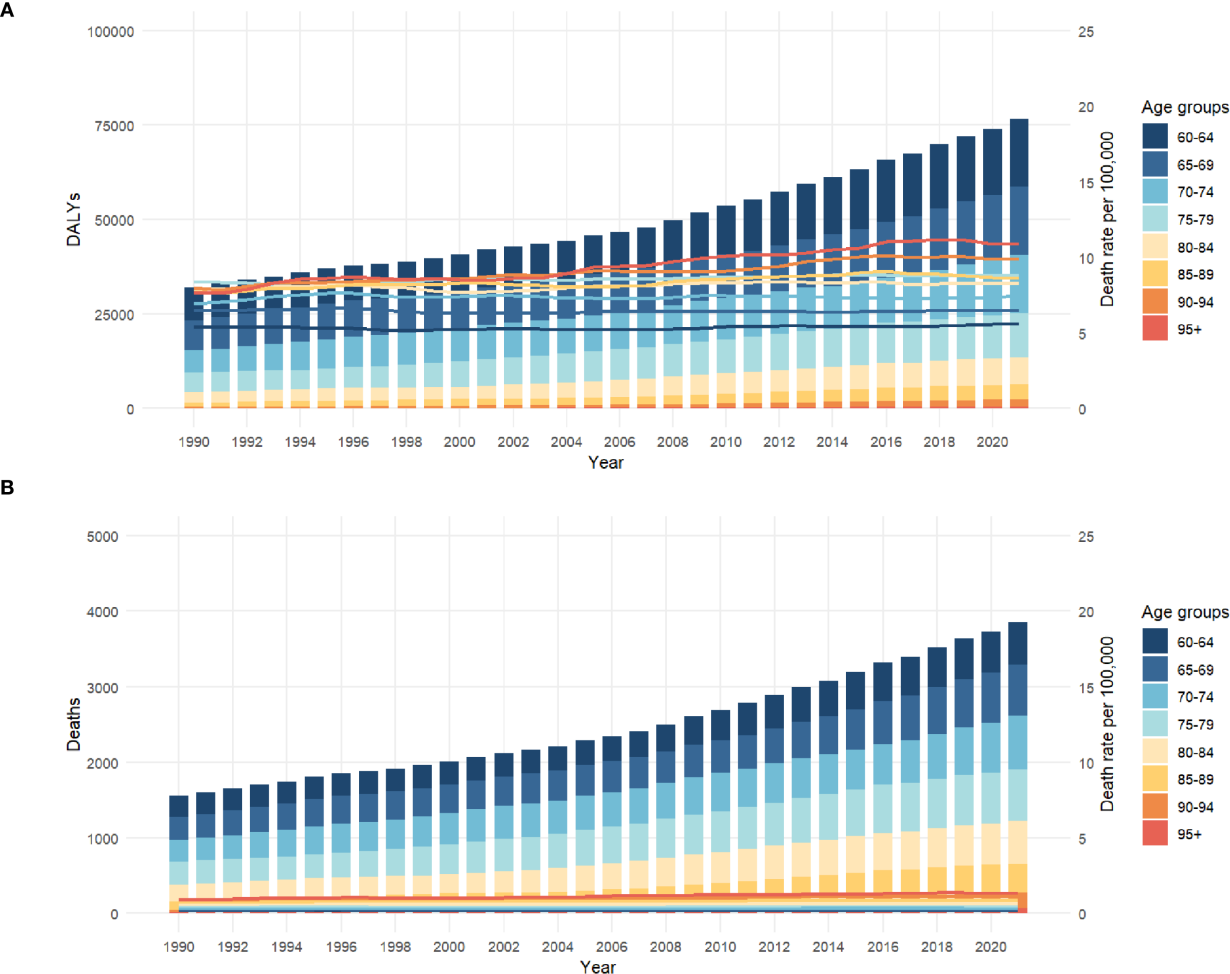
**(A)** shows the thyroid AS-DALYs in different age groups (1990-2021); **(B)** shows the thyroid ASMR in different age groups (1990-2021).

With the aging of the population, the death toll, mortality rate and disease burden are increasing year by year, especially for the group aged 85 and above. The disease burden (DALYs) of people over 70 years old is increasing year by year, especially those over 85 years old. In 2020, the burden will reach a high level, reflecting the increase of chronic diseases and senile diseases faced by the older adult. This poses a severe challenge to social medical care and health burden, especially to the very old group (**Figures 4A, B**).

**Figure 5:**
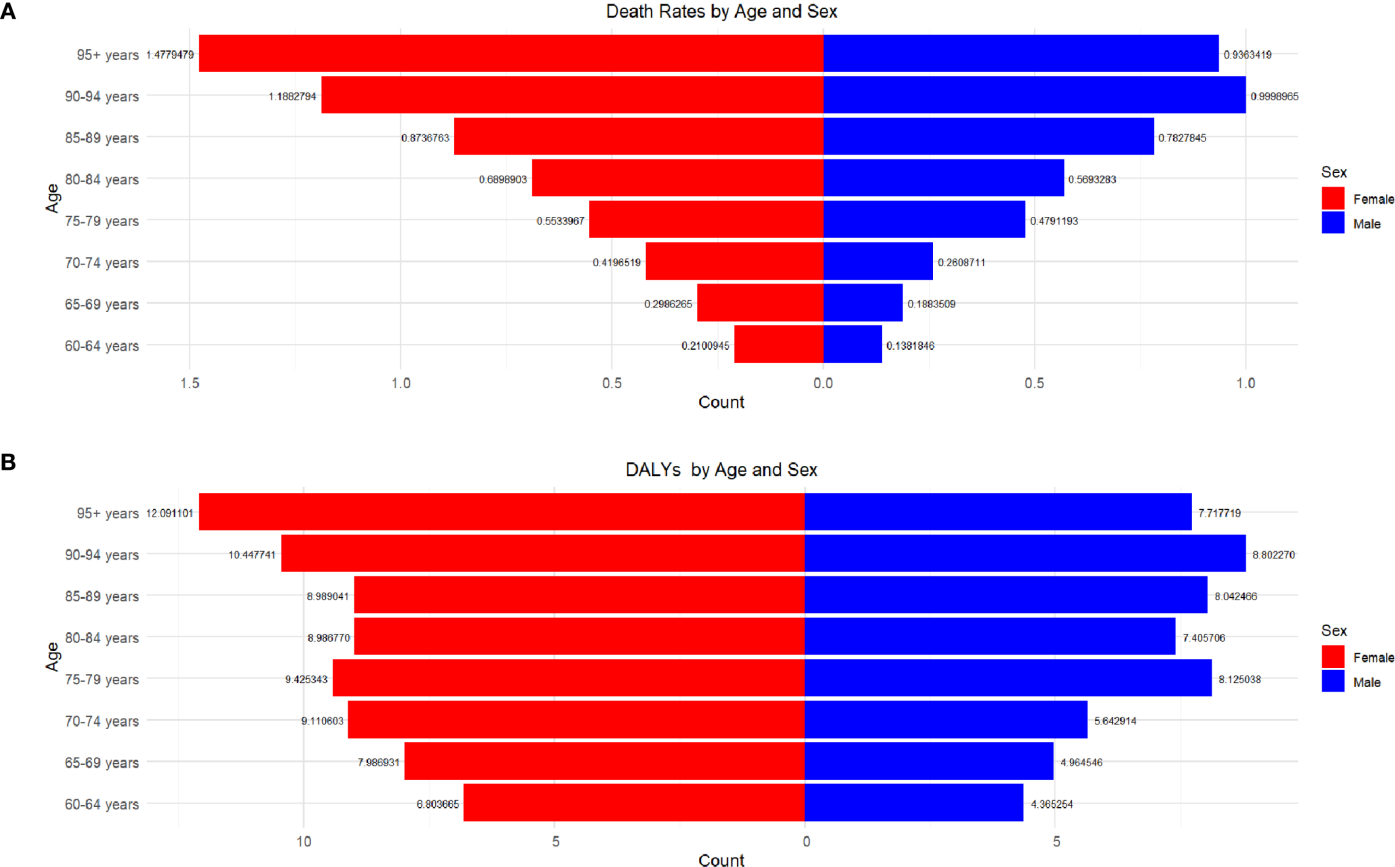
**(A)** shows the relationship between gender, age, and Death rate; **(B)** shows the relationship between gender, age, and DALYs.

At all ages, the mortality rate of women is always higher than that of men. For example, in the age group of 95 and above, the mortality rate of women is 1.4779 and that of men is 0.9363, indicating that the gender gap widens with the increase of age, especially in the older adult group. Although women live longer, the mortality rate of the older adult group is higher, and the gender gap between 60–69 years old and 65–69 years old is smaller (**Figure 5A**).

At the same time, with the increase of age, DALYs is on the rise, especially in the group over 95 years old. The DALYs of women are the highest (12.09) and men are also higher (7.72). Generally speaking, the DALYs of women are generally higher than that of men, especially in the group over 80 years old, and the gender gap is obvious. In the age group of 90-94, the DALYs of women is 10.45 and that of men is 8.80; In the 85–89 age group, the female is 8.99 and the male is 8.04. In addition, the gender difference gradually increases with age, especially after 75 years old, the DALYs of women increases significantly (**Figure 5B**).

### Prediction of disease burden of thyroid cancer in 2030

**Figure 6:**
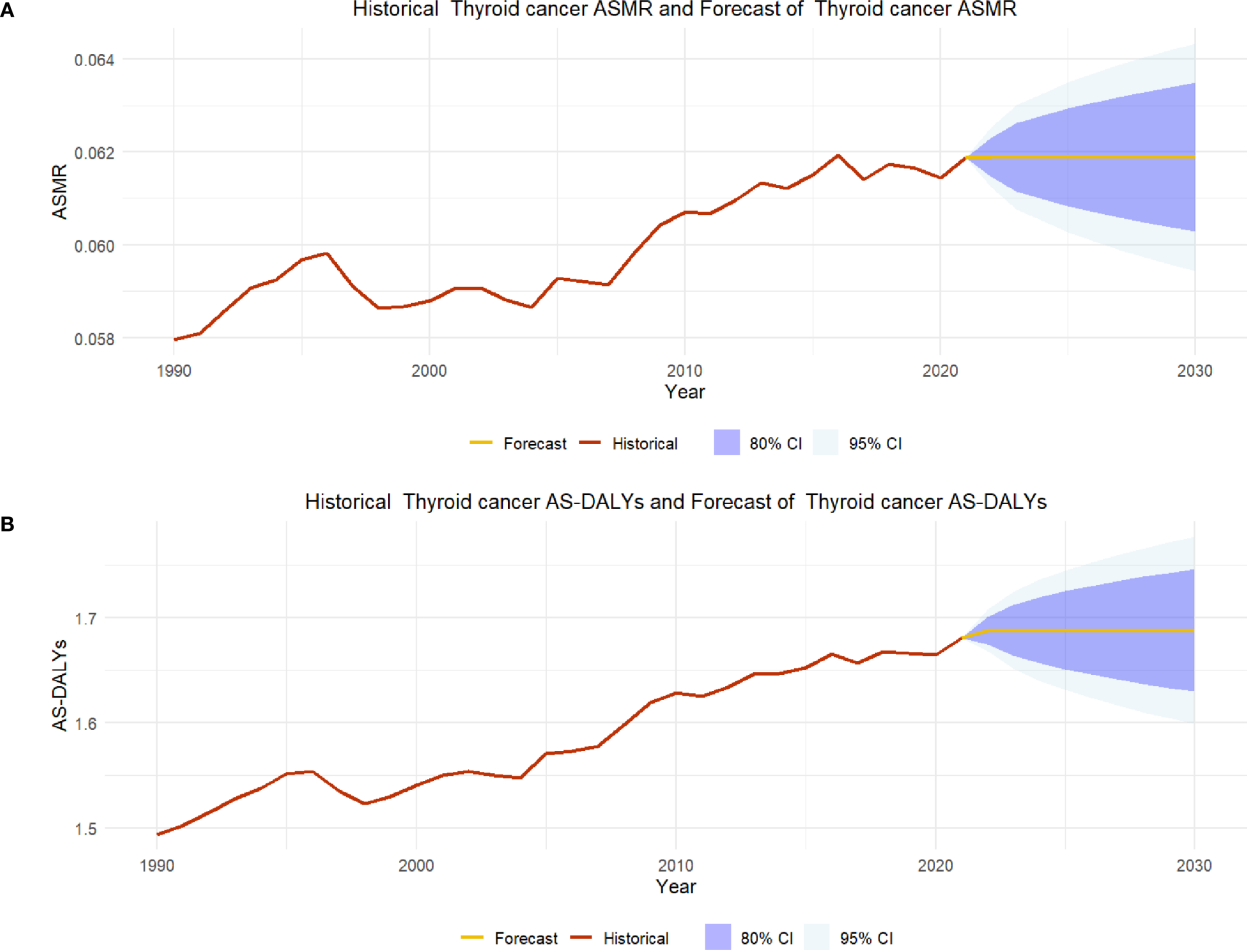
**(A)** shows the ASMR prediction results for thyroid cancer in 2030; **(B)** shows the ASMR prediction results for thyroid cancer in 2030.

Since 1990, the ASMR of thyroid cancer has experienced a slow increase, from about 0.058 to 0.062. According to the forecast, the mortality of thyroid cancer will remain relatively stable in the future, and it is predicted that by 2030, ASMR will remain at the level of about 0.062. Confidence intervals (80% CI: 0.060–0.063, 95% CI: 0.059–0.064) indicate that the mortality rate of thyroid cancer is narrow in the future, suggesting that the mortality rate of thyroid cancer may remain at the current level. From 1990 to 2020, the AS-DALYs of thyroid cancer also experienced a slight increase, from about 1.49 to 1.68. The prediction results show that AS-DALYs of thyroid cancer will be stable after 2020, and the value is expected to be close to 1.68 in 2030. The expansion of confidence interval (80% CI: 1.61–1.75, 95% CI: 1.59–1.76) reflects the consideration of future uncertainty. Overall, the disease burden of thyroid cancer may remain at the current level in the next decade (**Figure 6**).

## Discussion

Thyroid cancer, as a prevalent malignant tumor, has demonstrated a notable upward trend in both incidence and prevalence, particularly among the older adult population aged 60 and above, across the globe. This surge is closely linked to the global demographic shifts, advancements in medical technology, and the increased public health awareness (2, 18, 19). This paper will delve into the current situation, trend forecasts, regional disparities, and gender differences, while exploring the possible reasons behind the growing burden of thyroid cancer and the challenges faced in addressing it.

### Analysis of global trends in thyroid cancer mortality from 1990 to 2021

From 1990 to 2021, there has been a significant increase in the number of deaths from thyroid cancer worldwide, particularly among women, while the ASMR remains unchanged. This phenomenon deserves further exploration. The increase in the number of deaths from female thyroid cancer has not led to an increase in mortality rates, but rather shows a slight downward trend. Analyzing this trend can lead to several possible reasons. Firstly, the age structure of the female population has changed, and with the intensification of population aging, the proportion of older adult people is gradually increasing, which may be an important factor leading to a decrease in female mortality rates (20, 21) In addition, in recent years, the level of early diagnosis and treatment of thyroid cancer has improved, and the survival rate of female patients has significantly increased. This may also be one of the reasons why the mortality rate remains stable or slightly decreases (22–24).

However, the increase in deaths from male thyroid cancer shows a significant upward trend, and the ASMR has also increased. It is worth noting that the incidence rate of thyroid cancer in men has increased, which may be related to the fact that thyroid cancer has not received enough attention in the male population. In recent years, although the overall incidence rate of thyroid cancer has increased, the increase in mortality of thyroid cancer is still under control due to the diversity of cancer and the improvement of early diagnosis.

### Regional analysis of thyroid cancer mortality trends from 1990 to 2021

The observed increase in thyroid cancer deaths in low- to middle-SDI regions is a cause for concern and can be attributed to several critical factors. Late diagnosis is a significant contributor, as limited access to early screening programs and medical resources often results in patients being identified at more advanced stages of the disease, when treatment options are less effective. Additionally, the availability of essential treatments such as surgery and radioiodine therapy is often restricted in these regions due to financial constraints and inadequate healthcare infrastructure. This lack of access to effective treatments further exacerbates mortality rates. It is also possible that the distribution of thyroid cancer subtypes varies between high- and low-SDI regions, with more aggressive subtypes being more prevalent in the latter, contributing to the higher burden. Moreover, improvements in data collection and reporting systems in recent years may have led to a more accurate capture of previously underreported cases, thereby reflecting an apparent increase in the burden.

On the other hand, the slight decrease in thyroid cancer deaths in high-SDI regions indicates progress in managing the disease. This decline can be attributed to better treatment protocols, including advanced surgical techniques and targeted therapies, which have significantly improved patient outcomes. Effective screening programs in high-SDI regions play a crucial role by detecting thyroid cancer at earlier, more treatable stages. Furthermore, increased public awareness about thyroid cancer and its risk factors may have led to better preventive measures and lifestyle changes that reduce the incidence of the disease. Overall, these factors combined have contributed to the improved management and reduced mortality rates of thyroid cancer in high-SDI regions.

### Trend analysis of DALY for thyroid cancer from 1990 to 2021

From 1990 to 2021, the DALY of thyroid cancer worldwide has significantly increased, indicating an increasing disease burden. Although the annual average change rate of AS-DALY is relatively low, this long-term upward trend reveals that the health burden caused by thyroid cancer cannot be ignored globally, especially in low SDI regions where the disease burden is particularly evident. With the intensification of social aging, the burden of chronic diseases on the older adult population is increasing, and thyroid cancer, as an important disease, may further increase the global health burden in the future.

### Gender and age differences

Gender differences are particularly evident in the mortality rate and disease burden of thyroid cancer. It is widely recognized that the incidence rate of thyroid cancer is higher among females, though the reasons behind this disparity are not fully understood. One prominent hypothesis is the role of hormones, particularly estrogen, which is known to influence thyroid cell growth and differentiation. Studies suggest that estrogen receptors are present in thyroid tissue, and estrogen may promote thyroid cell proliferation, potentially leading to a higher incidence of thyroid cancer in females. Additionally, the menstrual cycle and pregnancy, which involve significant hormonal fluctuations, may also contribute to the increased risk of thyroid cancer in women. Another hypothesis is that females are more likely to undergo diagnostic scrutiny due to increased awareness and screening practices. Women generally have more frequent interactions with the healthcare system, which may lead to higher detection rates of thyroid cancer. This diagnostic bias could contribute to the observed higher incidence rates in females, though it does not fully account for the mortality differences. As age increases, the risk of thyroid cancer-related deaths in the female population escalates, particularly in the age group of 85 and above, where the mortality rate among women is significantly higher than that of men. This age-related disparity may be due to several factors, including the chronic nature of thyroid cancer and the medical resources required for long-term treatment. Older women may have more comorbidities, which can complicate treatment and increase mortality risk. Additionally, the long-term effects of thyroid cancer and its treatment may have a more significant impact on the health and quality of life of older women. Despite the relatively high mortality rate in the female population, the mortality rate of thyroid cancer in the male population is increasing rapidly. This trend may be attributed to several factors, including changes in diagnostic practices, increased exposure to risk factors, and potential underdiagnosis in the past. As awareness of thyroid cancer increases, more cases may be detected in males, leading to an apparent increase in mortality rates. Additionally, environmental and lifestyle factors, such as radiation exposure and smoking, may play a role in the increasing mortality rates in males. As women age, their DALY (Disability-Adjusted Life Years) is significantly higher than that of men, indicating a heavier health burden for women in their later years. This increased burden may be closely related to the chronic nature of thyroid cancer and the medical resources required for long-term treatment. The long-term management of thyroid cancer, including surgery, radiation therapy, and hormone replacement therapy, can have significant physical, psychological, and economic impacts on patients, contributing to the higher DALY scores observed in older women (25–27).

### Future predictions and challenges

According to the predicted results of the 2030 thyroid cancer mortality rate (ASMR) and AS-DALYs, the mortality rate and disease burden of thyroid cancer may remain relatively stable in the next decade. Despite the improvement in treatment levels and early screening techniques for thyroid cancer, the increasing trend of mortality and disease burden worldwide cannot be ignored, especially in low - and middle-income countries. In addition, with the intensification of population aging, especially among the older adult population aged 85 and above, the mortality rate and disease burden of thyroid cancer may show a further upward trend. In response to this phenomenon, countries around the world should pay more attention to the health issues of the older adult population and adopt more effective prevention and treatment strategies to alleviate the health burden caused by thyroid cancer.

### Current research deficiencies

The Global Burden of Disease (GBD) database is a crucial resource for global health research, yet it faces several limitations that can impact its effectiveness. The quality of data varies significantly due to its diverse sources, with low- and middle-income countries often contributing less reliable information, and updates can be delayed, which affects the timeliness of the data. The statistical models used in the GBD rely on certain assumptions that, if incorrect, could lead to inaccurate estimates. Additionally, the database may not fully cover rare or emerging health issues and may lack detailed analysis of specific health problems or the impact of certain risk factors. Limited resources can also affect the frequency of updates and the comprehensiveness of data collection and processing. Furthermore, the GBD data may not be sufficient for evaluating the effectiveness of specific health interventions or for capturing the full impact of health policies.

## Conclusion

The global mortality trend and disease burden of thyroid cancer exhibit complex patterns of change. Although the mortality rate in high SDI regions has decreased and the mortality rate in the male population has increased, overall, the mortality rate and disease burden of thyroid cancer are still on the rise globally. Gender, age, and regional differences are key factors, especially as the aging population intensifies, the burden of thyroid cancer may further increase in the future. Therefore, global public health policies should focus on improving early screening, enhancing diagnostic and treatment techniques, and promoting health education. Special attention should be paid to the insufficient medical resources in low SDI areas, and international cooperation and assistance should be promoted to narrow the health gap between regions.

## Data Availability

The original contributions presented in the study are included in the article/supplementary material. Further inquiries can be directed to the corresponding authors.
